# Sustained Malaria Transmission despite Reactive Screen-and-Treat in a Low-Transmission Area of Southern Zambia

**DOI:** 10.4269/ajtmh.20-0947

**Published:** 2020-11-23

**Authors:** Kelly M. Searle, Ben Katowa, Michael Musonda, Julia C. Pringle, Harry Hamapumbu, Japhet Matoba, Mukuma Lubinda, Timothy Shields, Tamaki Kobayashi, Jennifer C. Stevenson, Douglas E. Norris, Philip E. Thuma, Amy Wesolowski, William J. Moss, for the Southern and Central Africa International Center of Excellence for Malaria Research

**Affiliations:** 1 Division of Epidemiology and Community Health, University of Minnesota School of Public Health, Minneapolis, Minnesota;; 2 Macha Research Trust, Macha, Zambia;; 3 W. Harry Feinstone Department of Molecular Microbiology and Immunology, Johns Hopkins Bloomberg School of Public Health, Baltimore, Maryland;; 4 Department of Epidemiology, Johns Hopkins Bloomberg School of Public Health, Baltimore, Maryland

## Abstract

Malaria elimination strategies are designed to more effectively identify and treat infected individuals to interrupt transmission. One strategy, reactive screen-and-treat, starts with passive detection of symptomatic cases at health facilities. Individuals residing within the index case and neighboring households are screened with a malaria rapid diagnostic test (RDT) and treated if positive. However, it is unclear to what extent this strategy is effective in reducing transmission. Reactive screen-and-treat was implemented in Choma district, Southern Province, Zambia, in 2013, in which residents of the index case and neighboring households within 140 m were screened with an RDT. From March 2016 to July 2018, the screening radius was extended to 250-m, and additional follow-up visits at 30 and 90 days were added to evaluate the strategy. *Plasmodium falciparum* parasite prevalence was measured using an RDT and by quantitative PCR (qPCR). A 24-single nucleotide polymorphism molecular bar-code assay was used to genotype parasites. Eighty-four index case households with 676 residents were enrolled between March 2016 and March 2018. Within each season, parasite prevalence declined significantly in index households at the 30-day visit and remained low at the 90-day visit. However, parasite prevalence was not reduced to zero. Infections identified by qPCR persisted between study visits and were not identified by RDT. Parasites identified within the same household were most genetically related; however, overall parasite relatedness was low and similar across time and space. Thus, despite implementation of a reactive screen-and-treat program, parasitemia was not eliminated, and persisted in targeted households for at least 3 months.

## INTRODUCTION

Over the past two decades, there has been a decline in malaria incidence, morbidity, and mortality in more than 30 endemic countries, with eight formally transitioning from control to elimination strategies.^1,2^ However, a remaining challenge to elimination is identifying and treating all infected individuals (including asymptomatic and minimally symptomatic as well as symptomatic individuals) who may not seek care and treatment at health facilities but contribute to ongoing transmission.^3–5^ In low-transmission settings, these individuals may represent an important reservoir of transmission and present an obstacle to elimination.^5–8^ Strategies have been designed to identify and treat this reservoir through active case detection or mass drug administration.^9–12^ Active case detection strategies can be proactive, testing and treating individuals based on their membership in a specific risk group (e.g., school-age children),^6,12,13^ or reactive, by identifying transmission foci based on the diagnosis of symptomatic individuals.^14–16^ For reactive screen-and-treat, residents in the home of a confirmed symptomatic index case, and often their neighbors within a predefined radius, are screened with a rapid diagnostic test (RDT) and treated if positive.^14,16,17^ Reactive focal drug administration, by contrast, does not include screening, and instead, household residents are treated presumptively.^9,18,19^ An advantage of focal drug administration is that individuals with sub-patent infections, those below the limit of detection by an RDT, are treated. However, this strategy limits the program’s ability to accurately document the number of infected individuals within a community and requires a larger supply of antimalarials.

Zambia has had a dramatic decline in malaria cases in parts of the country over the past two decades, and Southern Province is considered a pre-elimination setting.^20^ Current (since 2013) surveillance and elimination strategies use volunteer community health workers (CHWs) to perform reactive screen-and-treat for confirmed index cases who have no history of travel outside the district.^14^ Members of the index and neighboring households within a 140-m radius are tested with an histidine-rich protein 2 (HRP-2)–based RDT, regardless of fever or other symptoms, within 1 week and treated if positive. However, it is unclear to what extent reactive screen-and-treat is effective in further reducing transmission and eliminating the parasite reservoir.^9,17,19,21–23^

To assess the spatial and temporal effectiveness of reactive screen-and-treat in a low-transmission setting within the catchment area of Macha Hospital in Choma district, Southern Province, Zambia, the screening radius was extended to 250 m, and follow-up visits were added at 30 and 90 days. The 250-m radius was chosen based on a previous study where 92% of cases were detected within this range.^19^ *Plasmodium falciparum* parasite prevalence was measured by quantitative PCR (qPCR) as well as RDT to detect low-level parasitemia. A 24–single nucleotide polymorphism (SNP) molecular bar-code assay was used to genotype parasites, calculate genetic relatedness over space and time, and measure genetic distance between successive or persistent infections.

## METHODS

### Study site and population.

This study was conducted in the rural catchment area of Macha Hospital in Choma district, Southern Province, Zambia, a low-transmission setting typical of southern Zambia. The region has a tropical savannah climate with a rainy season from December to April, followed by a cool dry season from May to August, and a hot dry season from September to November.^24^ The primary malaria vector is *Anopheles arabiensis*, which peaks during the rainy season, and malaria is almost exclusively due to *P. falciparum*.^25^ *Plasmodium falciparum* parasite prevalence measured by PCR declined from 9.2% in 2008 to 1% in 2013.^26^ Passively detected cases at health centers have since remained at historically low levels but with some variability, such as an increase in cases in 2016.^19,20^ Case management with artemisinin-based combination therapy was introduced in 2004, and insecticide-treated nets (ITNs) were widely distributed in 2007 and have been redistributed approximately every 3 years.^27,28^

### Study procedures.

Enrollment began in March 2016 and continued through March 2018, with follow-up visits continuing through July 2018. For this study, the reactive screen-and-treat radius was expanded from 141 to 250 m of the index case household. Community health workers who performed reactive screen-and-treat sent a short message service text message to the study team at Macha Research Trust when an eligible index case was identified. A study team member and the CHW visited the index household for a notification visit during which the head of household and residents were informed that the CHW and study team would return the following day to perform the combined programmatic reactive screen-and-treat and study procedures. GPS coordinates for the index household were collected at the notification visit and mapped in ArcGIS v. 10 (ESRI, Redlands, CA). A Geo-Eye-1 high-resolution satellite image collected in 2011 (Apollo Mapping, Boulder, CO) was used to identify neighboring households eligible for screening at 140- and 250-m radii from the index households. The images were printed and used to guide the CHW for programmatic reactive screen-and-treat within 140 m and the study team for enrollment of eligible households up to 250 m from the index household.

All residents of index and neighboring households within 250 m were eligible for study enrollment. Written informed consent was obtained from adults, and parental permission was provided for children. At the initial visit, a questionnaire was administered to collect information on demographic characteristics, malaria knowledge and prevention, recent malaria symptoms (e.g., fever, chills, and headache), health-seeking behaviors, recent malaria treatment, and recent travel history. Temperature was collected using a tympanic thermometer. Finger prick blood samples were collected for a *P. falciparum* HPR-2 RDT (SD Bioline, Gyeonggi-do, Republic of Korea) and as a dried blood spot (DBS) on a filter paper (Whatman 903^™^ Protein Saver Card, Sigma-Aldrich, Piscataway, NJ) for molecular analysis. Individuals with a positive RDT, with the exception of the index case on the initial visit, were offered treatment with Coartem^®^ (artemether plus lumefantrine, Novartis, Basel, Switzerland).

Study follow-up visits were conducted 30 and 90 days after the initial visit for all households. The study design was an open cohort, so residents in a household not present at a previous visit were invited to participate, offered informed consent, and enrolled at any follow-up visit. When enrolled households or participants were not present at the initial or follow-up visit, up to three visits were made. After these three attempts, if the household or participant was not available, the visit was treated as missing data. Similar study procedures were conducted at the follow-up visits as during the initial visit, with the exception that if the index case tested positive by RDT at a follow-up visit they were offered treatment with Coartem.

This study was approved by the Tropical Diseases Research Centre Ethics Review Committee and the Institutional Review Board at the Johns Hopkins Bloomberg School of Public Health.

### Laboratory procedures.

DNA was extracted from a single spot from the DBS card using a Chelex^©^ (Bio-Rad Laboratories, Hercules, CA) extraction protocol as previously described.^26^ Quantitative PCR was performed on all samples to detect the presence of the *P. falciparum* cytochrome b gene (*Pfcytb*). The limit of detection was determined to be one parasite per μL using standard genomic DNA dilution series and filter paper spotted with cultured parasites (NF54). Primers were designed to detect the presence of *Pfcytb*, and amplification was detected by fluorescence signal of SYBR^®^ Green. Each reaction contained 5 μL of DNA template, 5 μL of SYBR Green PCR Master Mix (Thermo Fisher, Waltham, MA), and 200 nM of forward primer (5′ CCT GAT AAT GCT ATC GTA 3′) and reverse primer (5′ TAA TAC AAT TAC TAA ACC AGC 3′). All qPCR-positive samples were evaluated on a 4% agarose gel to confirm the product size of the amplicon. Only samples confirmed by both qPCR and gel electrophoresis were considered qPCR positive.

A 24-SNP molecular bar-code assay was run using a TaqMan protocol at Macha Research Trust (see Supplemental Data). The 24-SNP molecular bar code was developed to distinguish parasite populations using PCR-based technology in a field research setting.^29^ DNA was extracted from a second DBS for samples confirmed to be parasite positive by qPCR. Because of low parasite DNA concentrations, samples were pre-amplified before performing the 24-SNP molecular bar-code assay. Specific methods for the pre-amplification step were described elsewhere.^30,31^ For each of the 24-SNP assays, 2.0 μL of pre-amplified sample DNA was added to 10 μL of TaqMan master mix, 7.5 μL distilled water, and 0.5 μL TaqMan commercially available primer and probe assay mixture.^29^ For each assay, three known positive controls and two negative, no-template controls were run. Positive controls consisted of DNA samples from *P. falciparum* strains obtained from BEI Resources (Manassas, VA) with known haplotypes for all 24 SNPs. Typically, 12-SNP assays were run for five samples at a time with controls on a 96-well plate. The assays were run on the Applied Biosystems StepOnePlus^™^ (Thermo Scientific), and the Roche LightCycler 480 II^™^ (Roche Diagnostics Corporation, Indianapolis, IN) real-time PCR systems. SNP calls were made automatically based on the high-resolution melt curves using software programs accompanying the real-time PCR systems as one of the two alleles or mixed. In cases where SNP calls could not be made automatically, determination was made manually by the study investigators but was not systematic by allele. Otherwise, the SNP call was classified as failed. Samples with failed SNP calls were repeated up to three times. If they failed on all repeated assays, they were treated as missing data.

### Statistical analyses.

Characteristics of the study population were compared by household type (index households, neighbors within 140 m, and neighbors between 141 and 250 m) and study visit. The malaria transmission season was defined as October through the following September and was used rather than the calendar year to more accurately capture the seasonal peak in cases and transmission. Differences in median age, distance from different stream orders (using the Strahler classification system, in which two category 1 streams join to form a category 2 stream, two category two streams join to form a category three stream, and so on), normalized difference vegetation index, and elevation were estimated using a Kruskal–Wallis test. Differences in gender, presence of fever (defined as tympanic temperature > 38°C), reported ITN use, recent travel, parasite prevalence by RDT, and parasite prevalence by qPCR were estimated using a Clopper–Pearson binomial exact test.

### Parasite genetic analysis.

Parasite bar coding was attempted on all qPCR-positive samples (see Data File 1). The distribution of parasite densities for all qPCR-positive samples was graphed and compared for samples that were successfully and not successfully bar-coded. Samples missing > 11 of the 24 SNPs in the bar code were excluded from analysis. Pairwise agreement between all samples was calculated using a modified SNP Π to account for mixed infections and missing data.^30^ Details of the calculation of the modified SNP Π were described elsewhere.^32^ In brief, this was calculated as the pairwise percent agreement between the 24-SNP sequences for each of the samples.^32^ This resulted in a pairwise genetic relatedness matrix. Pairwise time difference and distance between household matrices were created for all samples.

Genetic distance between bar-code sequences was also calculated for those with analyzable bar codes. Genetic distance between bar-code sequences was compared and graphed across household types and between participants who were parasitemic on multiple visits and was not set to a specified threshold. The genetic distance was compared between consecutive infections to determine relatedness between infections to infer persistence or new infections between visits.

## RESULTS

### Characteristics of the study population.

**Figure 1.**
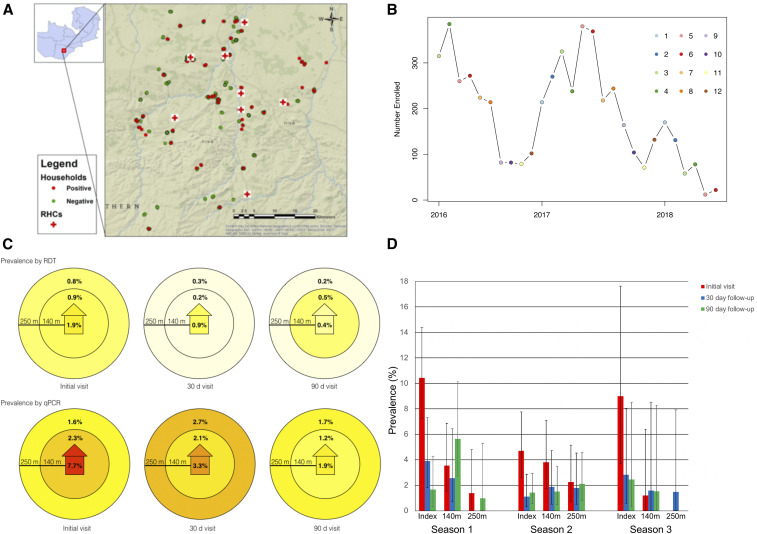
Study area and parasite prevalence of those sampled. (**A)** Location of all households sampled as part of the reactive screen-and-treat program within the catchment area of Macha Hospital within Choma district, Southern Province. Households are marked either positive (red) or negative (green) if at least one positive case (by quantitative PCR [qPCR]) was identified in that household. (**B**) Number of individuals enrolled in the study colored by month (1–12). (**C**) Parasite prevalence by rapid diagnostic test (RDT) (top row) and qPCR (bottom row) for the index household, households within 140 m, and those between 141 and 250 m. Tests were performed during initial, 30-day, and 90-day visits. Overall prevalence by RDT was lower than that by qPCR and highest in the index household. (**D**) Prevalence by season for the index, 140 m and 250 m households by visit number. This figure appears in color at www.ajtmh.org.

Eighty-four index households with 676 residents were enrolled and had an initial visit between March 2016 and March 2018 (Figure 1A). Enrollment decreased to 76 households with 544 residents on the 30-day visit and 75 households with 463 residents on the 90-day visit. One hundred and forty households with 675 residents were enrolled at the initial visit in neighboring households within 140 m of the index household, which decreased to 106 households with 438 residents and 111 households with 400 residents at the 30- and 90-day visits, respectively. One hundred forty-eight households with 864 residents were enrolled at the initial visit in neighboring households between 141 and 250 m of the index household, which decreased to 113 households with 574 residents and 99 households with 470 residents with follow-up data at the 30- and 90-day visits, respectively (Table 1).

**Table 1:** Characteristics of the study population by household type and visit

	Index households			Neighboring households within 140 m of the index household			Neighboring households within 141 to 250 m of the index household		
	Initial visit	30-day	90-day	Initial visit	30-day	90-day	Initial visit	30-day	90-day
Households	84	76	75	140	106	111	148	113	99
Individuals	676	544	463	675	438	400	864	574	470
Age (years)	12 (6–26)	12 (6–27)	11 (6–24)	12 (6–28)	12 (6–29)	12 (5–30)	12 (5–27)	12 (5–30)	12 (6–33)
Male (%)	46.8 (42.9–50.6)	46.4 (42.1–50.6)	44.8 (40.2–49.4)	46.3 (42.6–50.1)	45.2 (40.5–50.0)	45 (40.1–49.9)	44.5 (41.1–47.8)	46.1 (42.0 50.2)	46.3 (41.8–50.8)
Febrile (%)	20.3 (17.3–23.6)	10.4 (7.9–13.2)	5.7 (3.8–8.2)	15.3 (12.7–18.1)	11 ( 8.2–14.3)	5.4 (3.4–8.1)	15.2 (12.9–17.7)	8 (6.0–10.5)	8.3 (6.0–11.1)
Insecticide-treated nets use (%)	39.3 (35.4–43.2)	46.4 (42–50.8)	50.5 (45.7–55.2)	40.4 (36.5–44.4)	38.6 (33.7–43.7)	39.3 (34.2–44.6)	36.2 (32.8–39.7)	36.4 (32.2–40.8)	33.8 (29.3–38.5)
Recent travel (%)	9 (7.0–11.5)	4.9 (3.3–7.1)	4.7 (3.0–7.0)	3.5 (2.2–5.1)	3 (1.6–5.0)	4.2 (2.5–6.6)	5.9 (4.5–7.7)	4.1 (2.6–6.0)	4.6 (2.9–6.8)
Distance from a stream (km)									
First order	0.4 (0.2–1.0)	0.4 (0.2–1.0)	0.5 (0.2–1.0)	0.6 (0.2–1.1)	0.6 (0.2–1.1)	0.6 (0.2–1.1)	0.8 (0.4–1.0)	0.7 (0.4–0.9)	0.7 (0.4–1.0)
Second order	2.0 (1.2–2.7)	2.1 (1.2–2.8)	2.1 (1.2–3.0)	2.0 (1.1–2.9)	2.1 (1.1–2.9)	2.1 (1.2–2.9)	1.8 (1.0–2.5)	2.0 (1.2–2.6)	1.9 (1.2–2.6)
Third order	2.3 (1.1–4.2)	2.8 (1.2–4.4)	2.3 (1.2–4.4)	3.2 (1.0–5.9)	2.9 (1.0–5.2)	3.3 (1.0–5.9)	3.3 (0.9–6.1)	3.4 (1.0–6.1)	3.4 (1.3–6.2)
Fourth order	5.9 (2.9–10.9)	5.7 (2.7–11.5)	5.9 (3.5–11.5)	5.0 (2.2–8.0)	5.1 (2.3–7.8)	4.7 (1.8–7.8)	6.0 (3.7–10.3)	5.7 (2.0–10.4)	5.1 (2.0–9.2)
Fifth order	5.3 (1.6–10.5)	5.4 (1.6–10.9)	5.6 (1.2–11.1)	5.7 (0.9–10.8)	5.7 (0.8–9.0)	4.1 (1.0–10.5)	5.6 (21.7–8.7)	5.5 (1.6–7.4)	5.6 (2.0–8.7)
Sixth order	35.3 (25.9–46.8)	34.7 (25.9–44.3)	35.3 (27.0–44.3)	36.5 (31.6–50.9)	35.4 (27.1–48.3)	36.1 (31.5–48.9)	34.2 (25.9–40.7)	33.6 (25.4–36.4)	34.6 (25.4–40.6)
Normalized difference vegetation index	0.38 (0.35–40)	0.38 (0.35–0.40)	0.38 (0.35–0.40)	0.37 (0.34–0.40)	0.37 (0.35–0.40)	0.37 (0.35–0.40)	0.37 (0.34–0.39)	0.37 (0.34–0.40)	0.36 (0.34–0.38)
Elevation (m)	1,102 (1,056–1,142)	1,104 (1,056–1,154)	1,098 (1,061–1,138)	12,124 (1,095–1,146)	1,117 (1,087–1,136)	1,123 (1,095–1,138)	1,109 (1,058–1,136)	1,103 (1,054–1,128)	1,104 (1,054–1,138)
Rapid diagnostic test prevalence (%)	1.9 (1.0–3.3)	0.9 (0.3–2.1)	0.4 (0.05–1.5)	0.9 (0.3–1.9)	0.2 (0.006–1.3)	0.5 (0.06–1.8)	0.8 (0.3–1.6)	0.3 (0.04–1.2)	0.2 (0.005–1.1)
PCR prevalence (%)	7.9 (6.0–10.2)	3.3 (2.0–5.1)	1.9 (0.9–3.6)	2.3 (1.3–3.7)	2.1 (1.0–3.9)	1.2 (0.4–2.9)	1.6 (0.9–2.7)	2.8 (1.6–4.5)	1.7 (0.7–3.2)

A map of the participating households showed clustering due to the study design (Figure 1A). The study population was young, with a median age of 12 years for all household types and visits, with the exception of index households at the 90-day visit for which the median age was 11 years. The prevalence of fever was low and decreased significantly after the initial visit in all household types. Reported ITN use was low and increased slightly after the initial visit, but only in index households. The prevalence of recent travel also was low but was significantly higher in index households on the initial visit (9% [7.0–11.5]) than in all other visits and household types. Environmental factors did not differ between index and neighboring households (Table 1).

Parasite densities for qPCR-positive samples were low, with a median of 17.4 (4.2–78.8) copies per μL. Samples with successful bar-code sequences had a median of 19.7 (3.6–100.0) copies/μL, and those not successfully bar-coded had a median of 15.3 (4.2–63.0) copies/μL. Although parasite densities were slightly higher among samples that were successfully bar-coded, this was not statistically significant, and the distributions overlapped (Supplemental Figure S1).

#### Reactive screen-and-treat using an RDT did not capture most parasitemic individuals.

*Plasmodium falciparum* prevalence as measured by both RDT and qPCR was low. The highest prevalence was in index households at the initial visit (1.9% [1.0–3.3]) and decreased in all household types after the initial visit (Table 1, Figure 1B). Unsurprisingly, parasite prevalence measured by qPCR was significantly higher than that by RDT in all household types at each visit, ranging from 2 to 10.5 times higher (Figure 1B). In general, the sensitivity of the RDT was low, identifying only 20% of qPCR-positive individuals.

#### Reactive screen-and-treat reduced parasite prevalence but did not achieve elimination.

Following reactive screen-and-treat, parasite prevalence by both RDT and qPCR declined at 30 and 90 days compared with the initial visit. The highest parasite prevalence was recorded in index households at the initial visit (7.9% [6.0–10.2]) and decreased in all household types over follow-up with the exception of the 30-day visit in neighboring households between 141 and 250 m of the index household (Table 1, Figure 1B). When stratified by transmission season, parasite prevalence by qPCR showed similar decreasing trends by household type and follow-up visits (Figure 1C). Over the three transmission seasons, parasite prevalence was consistently highest in index households, with variability in prevalence over follow-up visits in all neighboring households. Parasite prevalence reached zero only in neighboring households between 141 and 250 m from index households on the 30-day visit in 2016 as well as the initial and 90-day visits in 2018, but was above zero for all other household types and visits (Figure 1D).

### Few individuals were repeatedly positive with or without treatment.

**Figure 2.**
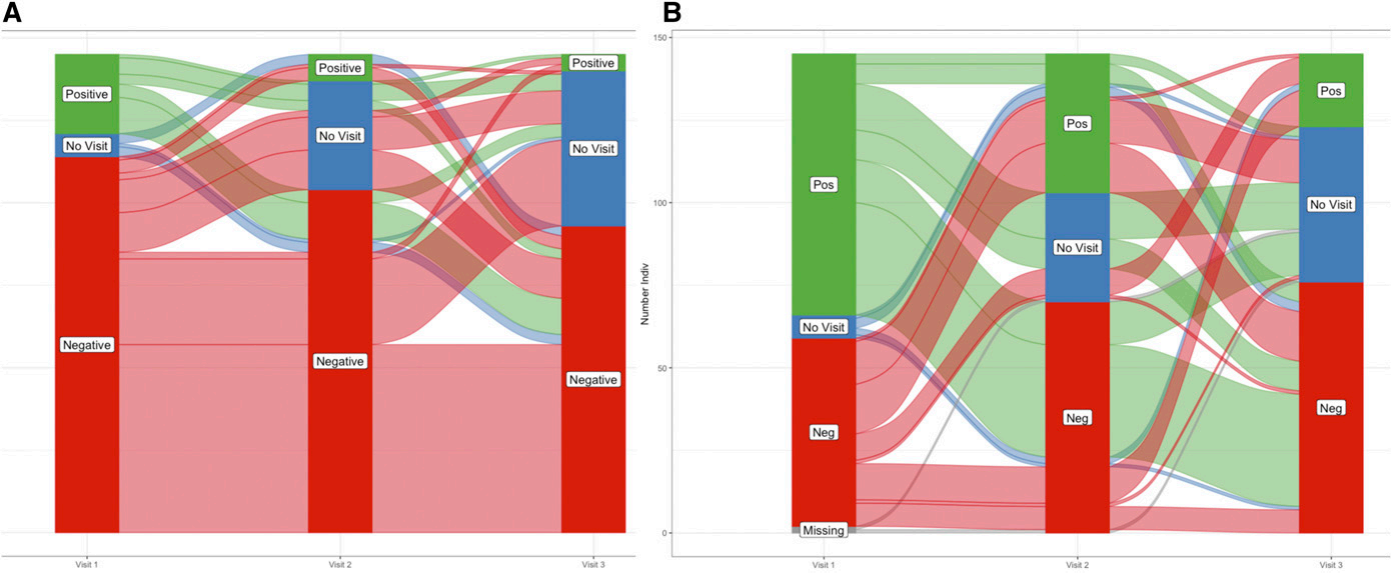
Trajectory of individual infection status spanning initial and subsequent visits. Individual test results by (**A**) rapid diagnostic test (RDT) and (**B**) quantitative PCR (qPCR) on the initial, 30-day, and 90-day visits for individuals who had at least one positive test (either by qPCR or RDT). Of these individuals, much fewer were RDT positive than those who were qPCR positive. However, in both instances, there were few consecutive positive events. This figure appears in color at www.ajtmh.org.

Only 145 participants had a positive RDT or qPCR result at any study visit. Thirty-seven participants (1.5%) had a positive RDT result with only one being repeatedly positive on a subsequent follow-up visit (initial visit and the 90-day follow-up) (Figure 2A). This individual had a missing 30-day follow-up visit. Although more individuals were parasitemic by qPCR (148 events among 138 participants), only 10 participants were positive by qPCR a second time (Figure 2B). Nine of these repeated qPCR-positive results were on the initial and 30-day visits with only one on the 30- and 90-day visits. None of these infections were identified by RDT on the initial visit, and only two were identified on the 30-day visit. Parasitemia identified on the 30-day visit by qPCR that persisted to the 90-day visit was also not identified on either visit by RDT. Although these individuals who were positive only by qPCR were not treated, most were negative by qPCR at the 30-day or 90-day visit without becoming RDT positive.

### Parasites were related across time and clustered in space.

**Figure 3.**
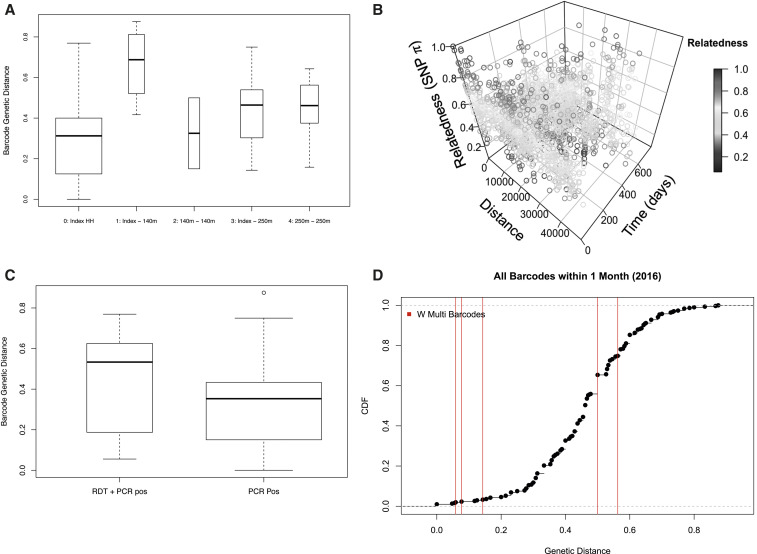
Genetic relatedness between infections. Genetic distance was calculated between infections using 24-single nucleotide polymorphism (SNP) bar codes. (**A**) Genetic distance of infections between all combinations of index household, households within 140 m, and households 141–250 m of the index household was calculated. In general, infections found within the same household were the most genetically related, with no clear pattern with increasing distances or within further distances (e.g., between infections all within 140 m of the index household). (**B**) Genetic distance between all pairs of infections by physical and temporal distance was calculated with more genetically related infections shown in red. (**C**) All infections were compared with the index infection, when possible, based on if the secondarily identified infection was rapid diagnostic test (RDT) and quantitative PCR (qPCR) positive or only qPCR positive. Surprisingly, infections that were only qPCR positive were more related to the index infection than those that were both RDT and qPCR positive. (**D**) For infections that occurred within 1 month of each other, the pairwise genetic distance was calculated. Few samples with multiple bar codes (shown in redlines) all occurred within 1 month of each other and the genetic distances are shown. Cumulative distribution function of all genetic distances for the within 1 month comparison is shown. This figure appears in color at www.ajtmh.org.

Ideally, a reactive screen-and-treat program would identify not only asymptomatic infections but also those related by transmission to the index case. Genetic relatedness based on 24-SNP bar-code data was used as a proxy for transmission relatedness. Only 68 samples (45.9%) had analyzable bar-code sequences from which genetic relatedness between infections was calculated, in part because of the low levels of parasitemia. Bar codes from parasites identified within the same household were most highly related (Figure 3A). However, there was no clear pattern between parasite relatedness in the index household and other households or between neighboring households. Relatedness decreased slightly with increasing time between detection (Figure 3B). However, parasites were related across seasons, as demonstrated by clustering of highly related parasites in three bands indicating the three transmission seasons (Figure 3B), suggesting some focal within-season parasite relatedness and maintenance of transmission with related parasites between seasons.

Interestingly, parasites were less related between an index case and a subsequent asymptomatic case identified as part of the reactive case detection event if the subsequent case was RDT positive (average genetic distance between index and RDT + & PCR + = 0.45, index and PCR+ = 0.32, *P* = 0.05), although this result was not significant (Figure 3C). This suggests that reactive screen-and-treat may not be capturing transmission-related infections.

### A small number of persistent infections may impact transmission.

Although the sample size was limited, the genetic distance between bar codes from individuals who had multiple qPCR-positive visits was analyzed. Only 10 individuals were qPCR positive on multiple visits. These 10 individuals resided in nine households and were collected during the 2016 transmission season. Of these 10 individuals, five had samples that were successfully bar-coded at two time points one month apart (no individuals with three PCR positive visits had three successful bar codes). For these five individuals, the genetic distance between these bar codes was calculated, and, for comparison, the genetic distance between bar codes collected 1 month apart also was estimated. Three individuals had bar codes that were highly related and above the expected genetic relatedness (Figure 3D). These three individuals were all sampled in March of 2016 and were children (ages 1, 5, and 15 years). Although the sample size was small for any statistical or robust comparisons, these results show evidence of persistent infections remaining undetected, as well as potentially new infections occurring within 1 month of reactive screen-and-treat.

## DISCUSSION

Reactive screen-and-treat has been shown to be operationally and logistically challenging, and costly.^19,23^ Nevertheless, reactive screen-and-treat is recommended by the WHO as an elimination strategy where feasible in countries with low transmission^33–35^ and was designed to leverage the spatial and temporal clustering of malaria transmission. The programmatic goal is to identify the chronically infected reservoir that is potentially maintaining transmission in low-burden settings. This study showed that a reactive screen-and-treat program could identify asymptomatic infections within index case households and provides epidemiologic evidence of focal transmission, but it also highlights the limitations of reactive screen-and-treat because of the low sensitivity of RDTs and the inability to achieve elimination.

Parasite prevalence was highest in index households at the initial visit in each season and decreased on follow-up visits but never reached zero. In 2015, Pringle et al.^36^ used a similar study design in the same setting but without longitudinal follow-up, and found that parasites collected closer in space and time within a single season were highly related using a panel of 26 microsatellite markers. This study included longitudinal follow-up visits and was conducted across multiple seasons. However, the 24-SNP genotyping method used in this study had lower resolution than the microsatellite method.^36^ Nevertheless, these findings were consistent with earlier studies that showed parasites from infections closer in space and time were more related, indicating local and focal transmission. Importantly, this study also found that parasites were related between follow-up visits and over seasons, suggesting transmission is sustained locally by persistent low parasitemic infections. Although the sample size was small, on the individual level, there was evidence of infections persisting undetected and new infections occurring between study visits, indicating the potential for sustained ongoing transmission in the presence of reactive screen-and-treat.

Parasitemia was very low in nearly all infections, with 70% falling below the limit of detection of the RDT (approximately 100 parasites/μL) and with a median of only 19.6 parasites/μL. This may partly explain the persistence of low-level transmission and the failure to achieve elimination despite a well-functioning reactive screen-and-treat program. Although the program was implemented according to national guidelines, it did not function as an efficient surveillance system to detect asymptomatic infections and reduce transmission. Even in a low-transmission setting, reactive screen-and-treat is resource intensive and falls short of achieving elimination by failing to identify low parasitemic infections. It is possible that *Plasmodium falciparum* histidine-rich protein 2/3 (pfhrp2/3) deletions in the parasite population could contribute to the inability of the RDT to detect infections. In this area, *pfhrp2*, but not *pfhrp3*, deletions were detected in a small sample of discordent RDT-negative/PCR-positive infections.^37^ However, the low parasite densities are the most likely reason for the limitations of the RDT to detect asymptomatic infections. The low parasitemia also resulted in high missingness in the genetic data, with only 46% of samples with sufficient data for analysis. We used targeted pre-amplification of parasite DNA to overcome potential bias in analyzing only samples with higher parasite densities.^31^ However, the high missingness highlights the challenges in conducting genetic analyses in settings with low parasitemia. The genetic analysis indicated that parasites were related within index households, at initial visits, between visits, and between seasons, suggesting these low parasitemic infections were maintained and likely transmitted into the following season. These low parasitemic infections among the asymptomatic reservoir could be a substantial factor associated with ongoing malaria transmission in this pre-elimination setting. A reactive strategy should help identify foci of transmission, but when screening and treatment are based on low sensitivity RDTs, this strategy is unable to capture all parasitemic individuals. Therefore, reactive focal drug administration in index households may be more effective and efficient in eliminating transmission foci.

Many of these findings are consistent with prior findings using epidemiologic and genetic data to identify focal transmission and can inform the National Malaria Elimination Programme on how to efficiently deploy interventions to interrupt transmission and achieve malaria elimination.^9,10,12,21,22,38^ Many previous studies have focused on the spatial extent and methods for screening and timing of potential follow-up visits in programs.^21,23,39,40^ Reactive screen-and-treat is now being implemented across several sub-Saharan African countries using similar methods as in southern Zambia.^9,11,16^ These have led to improved surveillance and an identification of an increased number of parasitemic individuals but have yet to successfully reduce transmission or achieve elimination.^9,23,41^

Reactive screen-and-treat requires maintenance of a steady supply of large numbers of RDTs for screening and depends on community volunteers with other responsibilities.^19^ There are many operational challenges to follow-up in every index case, especially during the high transmission season, and not every resident is at home at the time of the home visits.^19^ Further efforts are needed to guide screening and increase the efficiency of reactive screen-and-treat strategies, including the use of environmental clues such as streams.^21^ However, even under optimal conditions, when most infections are asymptomatic, the low sensitivity of the RDT as a screening tool is the biggest challenge to the effectiveness of reactive screen-and-treat.^19,22^ Rapid diagnostic tests only identified 20% of all parasitemic individuals, which decreased over follow-up visits as parasite prevalence decreased, and is the most likely reason there transmission was not interrupted.

It is clear that malaria transmission is spatially and temporally focal, especially in pre-elimination settings. This study provides further evidence that index households represent high-risk foci for infection. This relationship can be used to identify and treat the asymptomatic reservoir, specifically through reactive focal drug administration in index households with collection of DBS for surveillance by more sensitive testing with qPCR. However, what remains is better understanding of the underlying sources of transmission within and outside these foci that are not explained by parasite relatedness or longitudinal analyses. Further investigation is warranted to fully understand these transmission dynamics and the elimination strategies built upon these findings.

## Supplemental figure

Supplemental materials

## References

[1] WHO, 2018 World Malaria Report, 2018. Geneva, Switzerland: World Health Organization.

[2] Gething PW 2016 Mapping *Plasmodium falciparum* mortality in Africa between 1990 and 2015*.* N Engl J Med 375: 2435–2445. 27723434 10.1056/NEJMoa1606701 PMC5484406

[3] Kobayashi T 2019 Characteristics of subpatent malaria in a pre-elimination setting in southern Zambia*.* Am J Trop Med Hyg 100: 280–286. 30526744 10.4269/ajtmh.18-0399 PMC6367603

[4] Bousema T Okell L Felger I Drakeley C, 2014 Asymptomatic malaria infections: detectability, transmissibility and public health relevance*.* Nat Rev Microbiol 12: 833–840. 25329408 10.1038/nrmicro3364

[5] Lindblade KA Steinhardt L Samuels A Kachur SP Slutsker L, 2013 The silent threat: asymptomatic parasitemia and malaria transmission*.* Expert Rev Anti Infect Ther 11: 623–639. 23750733 10.1586/eri.13.45

[6] Bousema T Griffin JT Sauerwein RW Smith DL Churcher TS Takken W Ghani A Drakeley C Gosling R, 2012 Hitting hotspots: spatial targeting of malaria for control and elimination*.* PLoS Med 9: e1001165. 22303287 10.1371/journal.pmed.1001165 PMC3269430

[7] Bjorkman A Cook J Sturrock H Msellem M Ali A Xu W Molteni F Gosling R Drakeley C Mårtensson A, 2017 Spatial distribution of falciparum malaria infections in Zanzibar: implications for focal drug administration strategies targeting asymptomatic parasite Carriers*.* Clin Infect Dis 64: 1236–1243. 28431115 10.1093/cid/cix136 PMC5399945

[8] Stresman GH 2015 Focal screening to identify the subpatent parasite reservoir in an area of low and heterogeneous transmission in the Kenya highlands*.* J Infect Dis 212: 1768–1777. 26019285 10.1093/infdis/jiv302

[9] Aidoo EK 2018 Reactive case detection of *Plasmodium falciparum* in western Kenya highlands: effective in identifying additional cases, yet limited effect on transmission*.* Malar J 17: 111. 29534709 10.1186/s12936-018-2260-2 PMC5851086

[10] Bridges DJ Miller JM Chalwe V Moonga H Hamainza B Steketee R Silumbe K Nyangu J Larsen DA, 2017 Community-led responses for elimination (CoRE): a study protocol for a community randomized controlled trial assessing the effectiveness of community-level, reactive focal drug administration for reducing *Plasmodium falciparum* infection prevalence and incidence in Southern Province, Zambia*.* Trials 18: 511. 29096671 10.1186/s13063-017-2249-0 PMC5667476

[11] Gerardin J Bever CA Bridenbecker D Hamainza B Silumbe K Miller JM Eisele TP Eckhoff PA Wenger EA, 2017 Effectiveness of reactive case detection for malaria elimination in three archetypical transmission settings: a modelling study*.* Malar J 16: 248. 28606143 10.1186/s12936-017-1903-z PMC5469005

[12] Gerardin J Bever CA Hamainza B Miller JM Eckhoff PA Wenger EA, 2016 Optimal population-level infection detection strategies for malaria control and elimination in a spatial model of malaria transmission*.* PLoS Comput Biol 12: e1004707. 26764905 10.1371/journal.pcbi.1004707 PMC4713231

[13] Ruktanonchai NW DeLeenheer P Tatem AJ Alegana VA Caughlin TT Zu Erbach-Schoenberg E Lourenço C Ruktanonchai CW Smith DL, 2016 Identifying malaria transmission foci for elimination using human mobility data*.* PLoS Comput Biol 12: e1004846. 27043913 10.1371/journal.pcbi.1004846 PMC4820264

[14] Larsen DA 2015 Malaria surveillance in low-transmission areas of Zambia using reactive case detection*.* Malar J 14: 465. 26586264 10.1186/s12936-015-0895-9 PMC4653936

[15] Larson BA Ngoma T Silumbe K Rutagwera MR Hamainza B Winters AM Miller JM Scott CA, 2016 A framework for evaluating the costs of malaria elimination interventions: an application to reactive case detection in Southern Province of Zambia Malar J 15: 408. 27515533 10.1186/s12936-016-1457-5 PMC4982323

[16] Littrell M Sow GD Ngom A Ba M Mboup BM Dieye Y Mutombo B Earle D Steketee RW, 2013 Case investigation and reactive case detection for malaria elimination in northern Senegal*.* Malar J 12: 331. 24044506 10.1186/1475-2875-12-331 PMC3848815

[17] Hustedt J 2016 Reactive case-detection of malaria in Pailin Province, Western Cambodia: lessons from a year-long evaluation in a pre-elimination setting*.* Malar J 15: 132. 26931488 10.1186/s12936-016-1191-z PMC4774174

[18] Medzihradsky OF 2018 Study protocol for a cluster randomised controlled factorial design trial to assess the effectiveness and feasibility of reactive focal mass drug administration and vector control to reduce malaria transmission in the low endemic setting of Namibia*.* BMJ Open 8: e019294. 10.1136/bmjopen-2017-019294 PMC5829876 29374672

[19] Searle KM 2016 Evaluation of the operational challenges in implementing reactive screen-and-treat and implications of reactive case detection strategies for malaria elimination in a region of low transmission in southern Zambia*.* Malar J 15: 412. 27527347 10.1186/s12936-016-1460-x PMC4986207

[20] Mharakurwa S Thuma PE Norris DE Mulenga M Chalwe V Chipeta J Munyati S Mutambu S Mason PR; Southern Africa ICEMR Team, 2012 Malaria epidemiology and control in southern Africa*.* Acta Trop 121: 202–206. 21756864 10.1016/j.actatropica.2011.06.012 PMC3214248

[21] Bhondoekhan FRP 2020 Improving the efficiency of reactive case detection for malaria elimination in southern Zambia: a cross-sectional study*.* Malar J 19: 175. 32381005 10.1186/s12936-020-03245-1 PMC7206707

[22] Deutsch-Feldman M 2018 Efficiency of a malaria reactive test-and-treat program in southern Zambia: a prospective, observational study*.* Am J Trop Med Hyg 98: 1382–1388. 29557330 10.4269/ajtmh.17-0865 PMC5953385

[23] Sturrock HJ Novotny JM Kunene S Dlamini S Zulu Z Cohen JM Hsiang MS Greenhouse B Gosling RD, 2013 Reactive case detection for malaria elimination: real-life experience from an ongoing program in Swaziland*.* PLoS One 8: e63830. 23700437 10.1371/journal.pone.0063830 PMC3658965

[24] Moss WJ Hamapumbu H Kobayashi T Shields T Kamanga A Clennon J Mharakurwa S Thuma PE Glass G, 2011 Use of remote sensing to identify spatial risk factors for malaria in a region of declining transmission: a cross-sectional and longitudinal community survey*.* Malar J 10: 163. 21663661 10.1186/1475-2875-10-163 PMC3123248

[25] Kent RJ Thuma PE Mharakurwa S Norris DE, 2007 Seasonality, blood feeding behavior, and transmission of *Plasmodium falciparum* by *Anopheles arabiensis* after an extended drought in southern Zambia*.* Am J Trop Med Hyg 76: 267–274. 17297034 PMC4152308

[26] Laban NM Kobayashi T Hamapumbu H Sullivan D Mharakurwa S Thuma PE Shiff CJ Moss WJ; Southern Africa International Centers of Excellence for Malaria Research, 2015 Comparison of a PfHRP2-based rapid diagnostic test and PCR for malaria in a low prevalence setting in rural southern Zambia: implications for elimination*.* Malar J 14: 25. 25888818 10.1186/s12936-015-0544-3 PMC4340619

[27] Sipilanyambe N Simon JL Chanda P Olumese P Snow RW Hamer DH, 2008 From chloroquine to artemether-lumefantrine: the process of drug policy change in Zambia*.* Malar J 7: 25. 18230140 10.1186/1475-2875-7-25 PMC2248595

[28] Steketee RW Sipilanyambe N Chimumbwa J Banda JJ Mohamed A Miller J Basu S Miti SK Campbell CC, 2008 National malaria control and scaling up for impact: the Zambia experience through 2006*.* Am J Trop Med Hyg 79: 45–52. 18606763

[29] Daniels R 2008 A general SNP-based molecular barcode for *Plasmodium falciparum* identification and tracking*.* Malar J 7: 223. 18959790 10.1186/1475-2875-7-223 PMC2584654

[30] Searle KM 2017 Distinct parasite populations infect individuals identified through passive and active case detection in a region of declining malaria transmission in southern Zambia*.* Malar J 16: 154. 28420399 10.1186/s12936-017-1810-3 PMC5395854

[31] Mharakurwa S Daniels R Scott A Wirth DF Thuma P Volkman SK, 2014 Pre-amplification methods for tracking low-grade *Plasmodium falciparum* populations during scaled-up interventions in Southern Zambia*.* Malar J 13: 89. 24618119 10.1186/1475-2875-13-89 PMC4007587

[32] Ippolito MM Searle KM Hamapumbu H Shields TM Stevenson JC Thuma PE Moss WJ; For The Southern Africa International Center Of Excellence For Malaria Research, 2017 House structure is associated with *Plasmodium falciparum* infection in a low-transmission setting in southern Zambia*.* Am J Trop Med Hyg 97: 1561–1567. 28820722 10.4269/ajtmh.17-0299 PMC5817773

[33] WHO, 2012 Disease Surveillance for Malaria Elimination: an Operational Manual. Geneva, Switzerland: World Health Organization.

[34] WHO, 2018 World Malaria Report. Geneva, Switzerland: World Health Organization.

[35] WHO Programme, 2017 A Framework for Malaria Elimination. Geneva, Switzerland: World Health Organization.

[36] Pringle JC 2018 Genetic evidence of focal *Plasmodium falciparum* transmission in a pre-elimination setting in southern province, Zambia*.* J Infect Dis 219: 1254–1263. 10.1093/infdis/jiy640 PMC6452320 30445612

[37] Kobayashi T 2019 The search for *Plasmodium falciparum* histidine-rich protein 2/3 deletions in Zambia and implications for *Plasmodium falciparum* histidine-rich protein 2-based rapid diagnostic tests*.* Am J Trop Med Hyg 100: 842–845. 30719965 10.4269/ajtmh.18-0859 PMC6447119

[38] Grossenbacher B Holzschuh A Hofmann NE Abdullah Omar KA Stuck L Shariff Fakih B Ali A Yukich J Hetzel MW Felger I, 2020 Molecular methods for tracking residual *Plasmodium falciparum* transmission in a close-to-elimination setting in Zanzibar*.* Malar J 19: 50. 31996210 10.1186/s12936-020-3127-x PMC6988349

[39] Searle KM Shields T Hamapumbu H Kobayashi T Mharakurwa S Thuma PE Smith DL Glass G Moss WJ, 2013 Efficiency of household reactive case detection for malaria in rural Southern Zambia: simulations based on cross-sectional surveys from two epidemiological settings*.* PLoS One 8: e70972. 23940677 10.1371/journal.pone.0070972 PMC3735521

[40] Nelli L Guelbeogo M Ferguson HM Ouattara D Tiono A N’Fale S Matthiopoulos J, 2020 Distance sampling for epidemiology: an interactive tool for estimating under-reporting of cases from clinic data*.* Int J Health Geogr 19: 16. 32312266 10.1186/s12942-020-00209-1 PMC7171748

[41] van Eijk AM 2016 What is the value of reactive case detection in malaria control? A case-study in India and a systematic review*.* Malar J 15: 67. 26852118 10.1186/s12936-016-1120-1 PMC4744450

