# Supplementary material for: Sustained Malaria Transmission despite Reactive Screen-and-Treat in a Low-Transmission Area of Southern Zambia
Source: Am J Trop Med Hyg. 2020 Nov 23;104(2):671–9. doi: 10.4269/ajtmh.20-0947 (PMC7866307; doi:10.4269/ajtmh.20-0947)
Supplement: Supplementary file 1 [file tpmd200947.SD1.pdf]

Supplemental Information

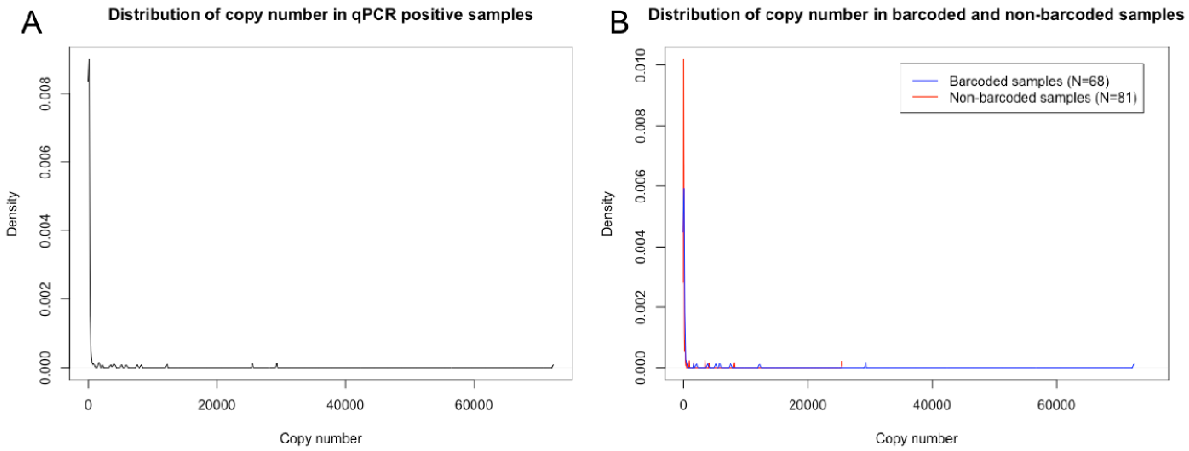

Figure S1: Distribution of parasite densities in: A) all qPCR positive infections and B) successfully and unsuccessfully barcoded qPCR positive infections.
